# Shifting Trends in Admission Patterns of an Acute Inpatient Psychiatric Unit in the State of New York

**DOI:** 10.7759/cureus.9285

**Published:** 2020-07-19

**Authors:** Bhumika Shah, Luba Leontieva, James L Megna

**Affiliations:** 1 Psychiatry and Behavioral Sciences, State University of New York Upstate Medical University, Syracuse, USA; 2 Psychiatry, State University of New York Upstate Medical University, Syracuse, USA

**Keywords:** admission patterns, inpatient unit, trends, psychiatry, hospital

## Abstract

Introduction

Deinstitutionalization has led to various changes in the utilization of healthcare services. The increased focus on treating patients within the community has led to variations in the utilization patterns of inpatient units. Shifts in demographic variables and disease-related, system-based, and economic factors have been observed. Due to the paucity of recent literature, this study was planned to assess the characteristics and treatment patterns in an acute inpatient psychiatric unit of a university hospital.

Methods

A retrospective observational study reviewing electronic medical records of patients in the context of demographic, disease-related, treatment-related, and system-based data was conducted over five years. Quantitative data were analyzed through descriptive statistics. Linear regression was used to study each variable across time.

Results

There was an increase in neurodevelopmental disorders (*p* = 0.024), substance use disorders (*p *= 0.041), and trauma and stressor-related disorders (*p *= 0.012), with a decrease in depressive disorders (*p *= 0.047). The use of restraints (*p *= 0.035) has increased significantly during the same period.

Conclusion

This study gives us an insight into the changing trends in patient characteristics which have the potential to inform the creation of improved services.

## Introduction

Inpatient units are important services for the care of the mentally ill. Previously, patients were primarily institutionalized to protect the community. With the improvement in treatment modalities and the belief that inpatient units were inhumane which was propagated by many including social activists, deinstitutionalization was introduced. The role of inpatient units is now to treat rather than segregate patients. In the period from ‘88 to ‘97, admissions for all diseases decreased by 8%. During the same period, admissions in general psychiatry institutes decreased, but a rise in admissions in the community hospitals by 18% was observed [1]. Community-based management has various benefits including reducing stigma, being more accessible for patients, and having better patient adherence [2]. Parallel to this, there has been a shift of treatment within the outpatient setting as well as acute care hospitals. Increased awareness, easy access, and increased availability have led to an escalated utilization of psychiatric care [3]. As biological interventions evolved and changes in the economics of healthcare, the length of stay in the inpatient units has decreased. Admission to an inpatient unit is generally reserved for acute care and severe illnesses.

With the changing trends, there is a growing interest in understanding the characteristics of the inpatients with the view to improve the efficiency and quality of the treatment centers [4]. This has led to investigations of various characteristics that impact admission. These range from demographics (age, gender, marital status, occupational status ), disease-related (diagnosis, medical comorbidities, comorbid substance use disorder, clinical outcome ), treatment-related (admission type, duration of admission, use of seclusion and restraints, number of admissions) to system-based factors (discharge disposition, type of insurance). 

From the ’80s through ’90s, there was a decline in admissions in patients over 64 years and a rise in admission for younger adults [1]. The utilization of hospital inpatient services is equal in both genders [5]. The majority of the patients are unemployed and without a partner [6-7]. Over time, the diagnosis of patients being admitted has varied. An earlier study that looked at the period in 1988-97 reported increasing discharges from community hospitals for patients who suffer from schizophrenia and affective disorders while a decline in those suffering from anxiety and related disorders [1]. In the early 21st century, the top four diagnostic groups that were admitted were depression (29%), bipolar disorder (22%), schizophrenia (18%), and substance use disorders (14%) [8]. By 2015, research revealed the maximum number of patients who utilize the inpatient unit suffers from three disorders. In decreasing order of frequency, these were personality disorders, major depressive disorder, and substance use disorder [5]. Besides alteration in diagnosis, the average length of inpatient admission in the United States has also changed. The duration of admission in the majority of the general hospitals and a few private hospitals has been reported to range from 6.8 to 95.4 days [9]. Over time, there has been a determined effort to decrease the use of restraints and seclusions in acute care [10].

The characteristics and trends of admission in the previous two decades have been researched but there is a paucity of recent literature regarding the same. This study was planned to assess the demographic, disease-related, treatment-related, and system-related characteristics of patients admitted in an acute inpatient psychiatry unit in the state of New York. We hypothesize an upward trend in the number of patients suffering from personality, substance-related and trauma-related disorders along with increased use of seclusions and restraints as a result of increased aggression in the admitted patients.

## Materials and methods

Participants

This is a retrospective observational study reviewing electronic medical records of patients admitted to an acute psychiatric unit of an academic hospital located in Upstate New York. The hospital caters to a community with a population of about 147,000 people. The acute psychiatric unit admits patients within the hospital such as the Emergency Department and other medical floors, and from other neighboring medical facilities. The study consisted of patients admitted between April 1, 2014 and April 31, 2019. The study was approved by the Institutional Review Board of the Upstate Medical University and was carried out per the latest version of the Declaration of Helsinki.

Procedure

Demographic and clinical variables were collected via chart review. The patients were between 18 and 89 years of age. The variables collected were: age, gender, marital status, employment, number of admissions, length of stay, use of restraints and seclusion, discharge disposition, and diagnosis.

Statistical analysis

The quantitative data were analyzed through descriptive statistics. The data were presented as mean ± standard deviation (SD), median with interquartile range (IQR), or percentages. Bivariate analyses using contingency tables (*χ*^2^) were performed between the variable to determine any significant association. Linear regression was used to study each variable across time. An alpha value (p-value) less than 0.05 was considered significant.

## Results

The psychiatric unit is a 24-bedded ward in an academic facility that accepts both involuntary and voluntary patients. A total of 2984 patients were admitted over five years.

Demographics

The mean age of patients was 40.33 years (median 40, range 18-91 years). The most common age group of patients being admitted was between 36 and 55 years. Compared to females, males were admitted at a marginally higher rate (51.97%). Over 80% of the inpatients were either single, widowed, or divorced. Unemployment was present in 42.90% of the patients and 30.63% were disabled due to either a chronic mental illness, physical or medical disability. Nearly a quarter of patients were admitted more than once. There was no change in these variables across time (Table 1).

**Table 1:** Demographics of the patients admitted over the five-year period

	Year 1 (n, %)	Year 2 (n, %)	Year 3 (n, %)	Year 4 (n, %)	Year 5 (n, %)	Total (n, %)	P-value
Age							
18-35 years of age	130 (10.63)	254 (20.78)	288 (23.56)	261 (21.35)	289 (23.64)	1222 (40.96)	0.12
36-55 years of age	200 (15.82)	259 (20.49)	302 (23.89)	253 (20.01)	250 (19.77)	1264 (42.35)	0.5
More than 55 years of age	74 (14.85)	112 (22.48)	115 (23.09)	90 (18.07)	107 (21.48)	498 (16.68)	0.5
Gender							
Male	218 (14.05)	346 (22.3)	349 (22.5)	312 (20.11)	326 (21.01)	1551 (51.97)	0.35
Female	186 (12.97)	279 (19.46)	356 (24.84)	292 (20.37)	320 (22.33)	1433 (48.02)	0.19
Marital Status							
Single, widowed or divorced	335 (13.81)	519 (21.40)	548 (22.59)	486 (20.04)	537 (22.14)	2425 (81.26)	0.21
Married	65 (12.1)	98 (18.24)	151 (28.11)	117 (21.78)	106 (19.73)	537 (17.99)	0.38
Unspecified	4 (18.18)	8 (36.36)	6 (27.27)	1 (4.54)	3 (13.63)	22 (0.73)	0.36
Employment							
Student	4 (4.59)	20 (22.98)	18 (20.68)	20 (22.98)	25 (28.73)	87 (2.91)	0.07
Unemployed	194 (13.66)	299 (21.05)	333 (23.45)	273 (19.22)	321 (22.6)	1420 (47.58)	0.23
Disabled	132 (14.44)	203 (22.21)	203 (22.21)	206 (22.53)	170 (18.59)	914 (30.63)	0.52
Employed	69 (12.66)	94 (17.24)	150 (27.52)	105 (19.26)	127 (23.30)	545 (18.26)	0.24
Unknown	5 (27.77)	9 (50)	1 (5.55)	0	3 (16.66)	18 (0.60)	0.31
Admissions							
Once	301 (13.4)	462 (20.56)	538 (23.95)	448 (19.94)	497 (22.12)	2246 (75.26)	0.22
Twice or more	103 (13.95)	163 (22.08)	167 (22.62)	156 (21.72)	149 (20.35)	738 (24.73)	0.37

Disease-related characteristics

**Figure 1:**
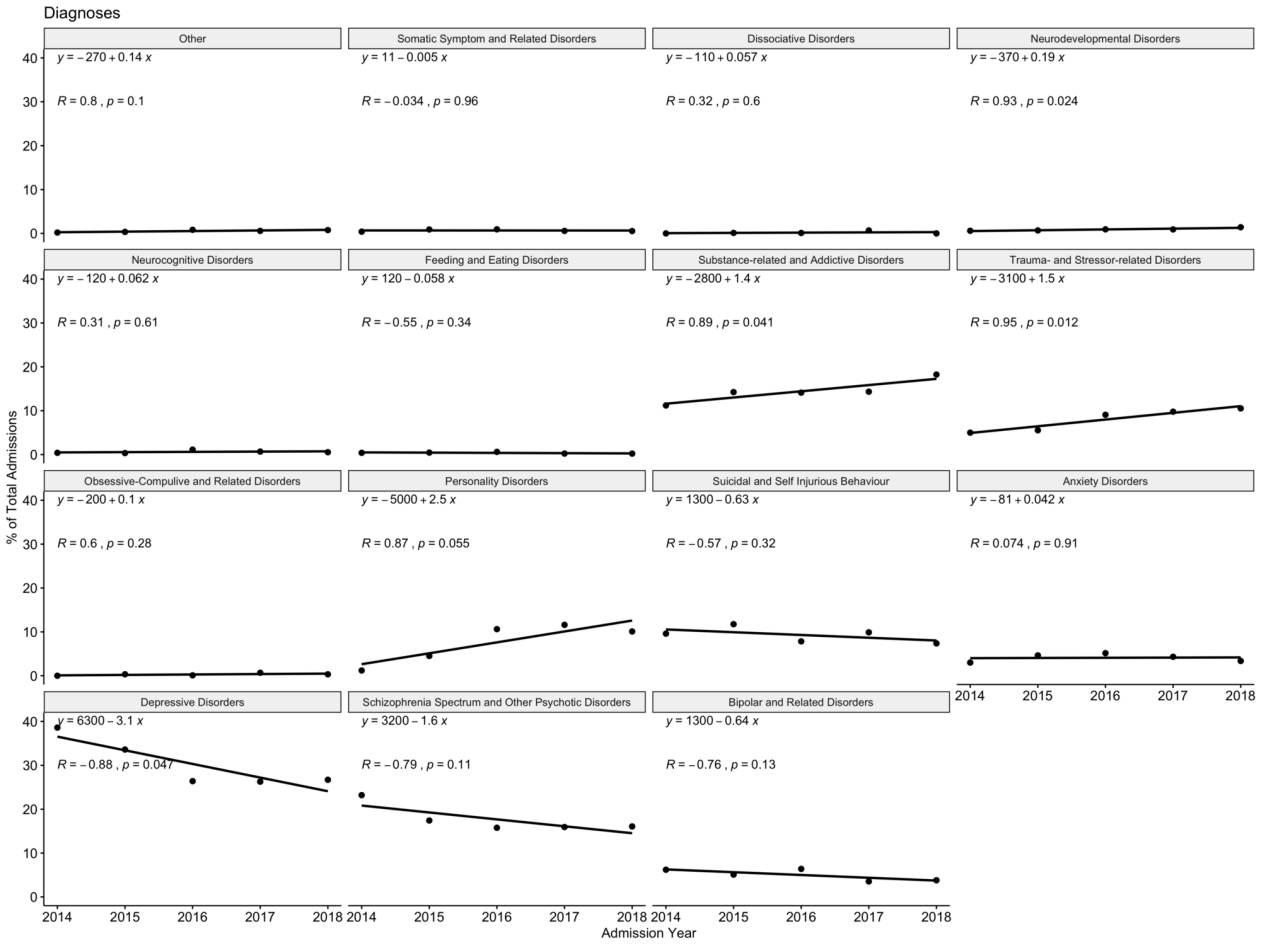
Trends in diagnosis over the five-year period

**Figure 2:**
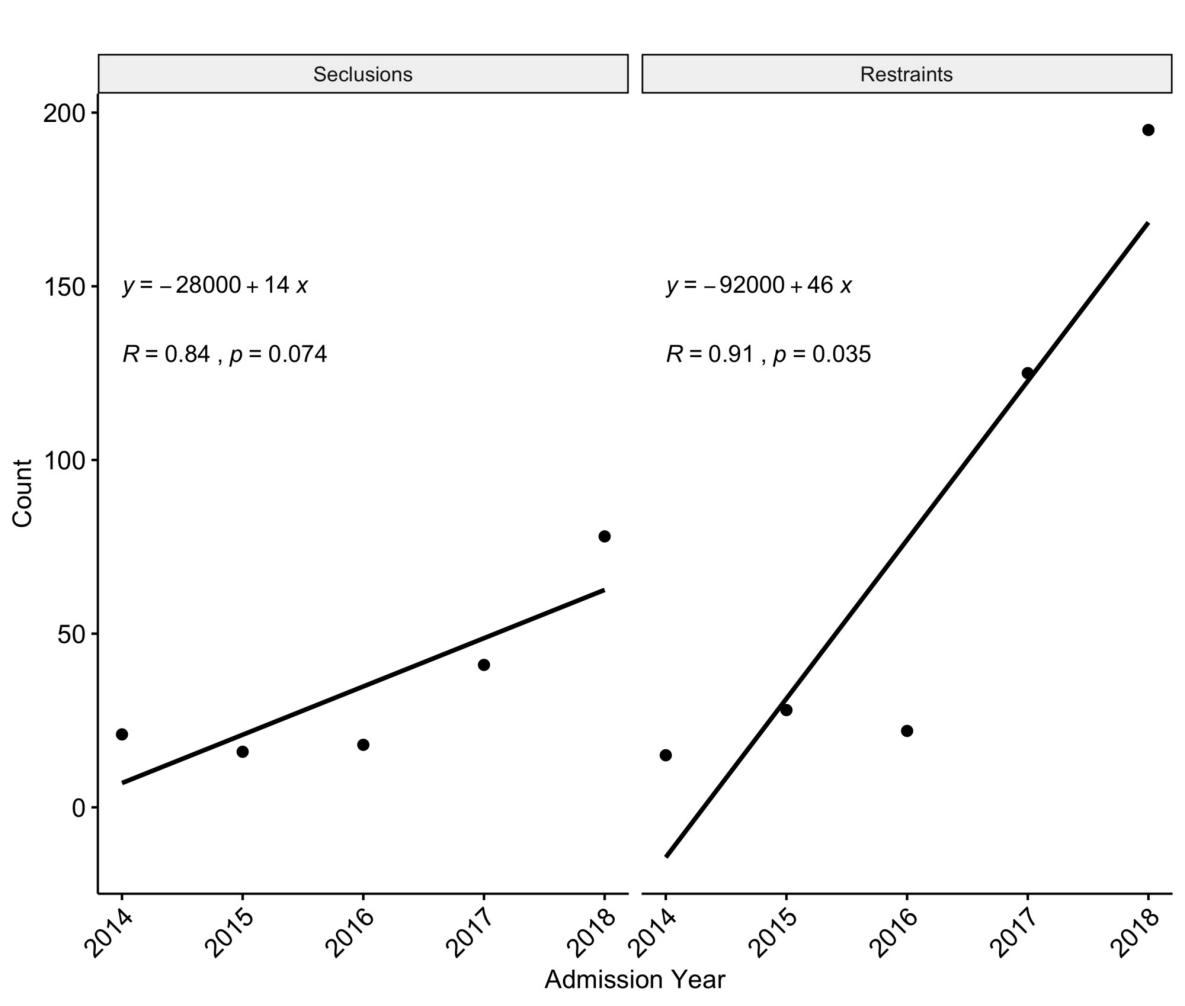
Changes in seclusions and restraints over the five-year period

The most frequent diagnosis seen in the patients was depressive disorder (29.44 %), followed by schizophrenia spectrum and other psychotic disorders (17.12%) and substance-related and addictive disorder (14.76%; Figure 1). Over the 5 years, there was an increase in patients admitted with neurodevelopmental disorder (*p *= 0.024), substance-related and addictive disorder (*p *= 0.041), and trauma and stressor-related disorders (*p *= 0.012). There was a nearly significant increase in personality disorders (*p* = 0.055). On the other hand, there was a statistical decline in patients admitted with depressive disorders (*p *= 0.047; Figure 2).

There has been a significant rise in the use of four-point restraints over time (Figure 2). Similarly, there has been an increase in patients being discharged to another facility (Table 2). The most frequent discharge disposition is being discharged home or self-care. The length of stay has not shown variation, ranging from 1 to 136 days, with the median length being 5 days (IQR 3-8 days).

**Table 2:** Treatment and System-based characteristics of the patients over the five-year period

	Year 1 (n, %)	Year 2 (n, %)	Year 3 (n, %)	Year 4 (n, %)	Year 5 (n, %)	Total (n, %)	P-value
Seclusions	21 (12.06)	16 (9.19)	18 (10.34)	41 (23.56)	78 (44.82)	174 (5.83)	0.07
Restraints	15 (3.89)	28 (7.29)	22 (5.71)	125 (32.46)	195 (50.64)	385 (12.9)	0.03
Length of stay							
0-10 days	302 (12.26)	498 (20.21)	616 (25.01)	505 (20.50)	542 (22)	2463 (82.54)	0.25
11-20 days	71 (18.83)	85 (22.54)	76 (20.15)	71 (18.83)	74 (19.62)	377 (12.63)	0.72
More than 20 days	31 (21.52)	42 (29.16)	13 (2.08)	28 (19.44)	30 (20.83)	144 (4.82)	0.70
Discharge Disposition							
Left against medical advice	3 (33.33)	1 (11.11)	2 (22.22)	3 (33.33)	0	9 (0.30)	0.42
Discharged to another facility	33 (13.04)	46 (18.18)	51 (20.15)	64 (25.29)	59 (23.32)	253 (8.47)	0.028
Home or self-care	361 (13.4)	574 (21.31)	649 (24.09)	532 (19.75)	577 (19.47)	2693 (90.24)	0.313
Other	6 (20.68)	4 (13.79)	4 (13.79)	5 (17.24)	10 (34.48)	29 (0.97)	0.31

## Discussion

This paper discusses the different characteristics of inpatients in a short-stay academic unit of a general hospital for over five years. The hospital accepts both involuntarily committed and voluntary patients. The majority of the patients are referred from the emergency department, while the rest arrive from the medical floors of the University hospital. Most demographic factors have remained constant and match prior literature. The age and gender of our patients were in keeping with past American and global studies [6-7,11]. Earlier studies report the mean age of patients in the psychiatric inpatient unit has been 43 years with increasing admission in the age groups of 20-64 years [1-2]. Both genders in our study are utilizing services with nearly the same frequency as depicted in prior literature [7]. It is known that a supportive marital relationship is protective against mental illness [12]. It may also be construed that this is the effect of the disorder. Similarly, unemployment either as a cause or a consequence is seen frequently in mentally ill patients. Our study follows similar trends. Admission more than once was reported in nearly one-quarter of patients. These results are similar to those reported by former researchers [7].

The disease factors have revealed changing trends. Depression, psychosis, and substance use disorder are the most cited diagnosis in our cohort. This is similar to most studies done in the past. Some report psychosis having a higher prevalence in inpatients whilst others report affective disorders [4,10]. Despite depression being the most common illness in our cohort, it has shown a decline over the five years. The decline in patients suffering from depression could be related to better treatment and services available within the community [1]. Substance use disorders, personality disorders, trauma-related disorders, and neurodevelopment disorders have revealed increasing trends in our study. Individuals with substance use disorders are known for non-adherence to treatment and comorbidities which lead to an increase in the utilization of inpatient services [13]. Personality disorders have been reported as the most common reason for admission in some studies [4-5]. In the period from 2000 to 2007, researchers have cited an increase in admissions from 6.1% to 9.6% in patients suffering from personality disorders [14]. Patients suffering from this disorder are known to have an increased risk of suicide, homicide, comorbidity, and somatic complaints [15]. This often leads to admission especially when community outpatient services cannot meet their needs. Violence is one of the common reasons for admission and is known to occur frequently with personality disorders, substance use disorders, and neurodevelopmental disorders such as intellectual disability [16-18]. As trauma increased in our lives, trauma-related disorders requiring short-term inpatient management also increased. Such results explain the increase in admission rates of patients with personality disorders, neurodevelopmental disorders, and substance use disorders.

In our study, the use of seclusion has shown no change over time. Seclusion is rarely utilized, and this is in keeping with the philosophy of the least restrictive treatment option to be used. Restraints have been reported to be utilized in 7.4% to 17 % of inpatients [19]. In our study, the use of restraints has shown a significant upward trend from the first year to the fifth year and a mean of 12.9%. Restraints are often used to curtail aggression seen in inpatients. With an increase in the number of patients having disorders leading to aggression such as substance use disorders and personality disorders, the use of restraints has increased in our context. The use of non-medicated methods could be implemented to improve the patients’ coping skills and decrease the use of restraints [20-21]. Debriefing the unit after the event and training of the doctors and staff in de-escalation could help further. An addition of a crisis response team that is trained in such events could benefit inpatient units tremendously. Having a large unit with single rooms may decrease aggression. Despite the need for restraints, the length of stay has remained constant over the study period. Our mean length of stay was 7.4 days with a median of five days. Previous studies from the same hospital reported a mean of 23.8 days in 2005 and 9 days in 2017 [5,22]. This decrease in the length of stay is a global phenomenon that is occurring due to improvement in treatment options and as cost-cutting measures by managed-care insurance companies. Our current findings match those reported in other community hospitals. [23, 24]. One other factor that could be related to the length of stay has been having a place to go after discharge. Our patients were most frequently discharged either to their homes or in self-care. The recent availability of community-based treatment options such as assertive community treatment and intensive case management has resulted in better management care [1,7].

Our sample is large and of the recent past helps in providing insight into the shifting tends of admission patterns. A few limitations of the study include a limited number of variables studied and the sample restricted to one acute inpatient unit.

## Conclusions

In conclusion, we notice that there are changing trends in disease-related, treatment-related, and system-based characteristics. While the length of stay has not changed over the five years, the frequency of patients being discharged to another facility has increased. Despite depression being the most common diagnosis, there is an upswing in other disorders such as substance use disorder, neurodevelopmental disorders, trauma-related disorders, and personality disorders. With an increase in disorders leading to aggression, there is an increase in the use of restraints. These findings should be utilized to improve inpatient services to cater to the changing trends.
